# Molecular basis of V-ATPase inhibition by bafilomycin A1

**DOI:** 10.1038/s41467-021-22111-5

**Published:** 2021-03-19

**Authors:** Rong Wang, Jin Wang, Abdirahman Hassan, Chia-Hsueh Lee, Xiao-Song Xie, Xiaochun Li

**Affiliations:** 1 Department of Molecular Genetics, University of Texas Southwestern Medical Center, Dallas, TX USA; 2 Eugene McDermott Center for Human Growth and Development, University of Texas Southwestern Medical Center, Dallas, TX USA; 3 Department of Structural Biology, St. Jude Children’s Research Hospital, Memphis, TN USA; 4 Department of Biophysics, University of Texas Southwestern Medical Center, Dallas, TX USA

**Keywords:** Membrane proteins, Target validation, Cryoelectron microscopy

## Abstract

Pharmacological inhibition of vacuolar-type H^+^-ATPase (V-ATPase) by its specific inhibitor can abrogate tumor metastasis, prevent autophagy, and reduce cellular signaling responses. Bafilomycin A1, a member of macrolide antibiotics and an autophagy inhibitor, serves as a specific and potent V-ATPases inhibitor. Although there are many V-ATPase structures reported, the molecular basis of specific inhibitors on V-ATPase remains unknown. Here, we report the cryo-EM structure of bafilomycin A1 bound intact bovine V-ATPase at an overall resolution of 3.6-Å. The structure reveals six bafilomycin A1 molecules bound to the c-ring. One bafilomycin A1 molecule engages with two *c* subunits and disrupts the interactions between the c-ring and subunit *a*, thereby preventing proton translocation. Structural and sequence analyses demonstrate that the bafilomycin A1-binding residues are conserved in yeast and mammalian species and the 7’-hydroxyl group of bafilomycin A1 acts as a unique feature recognized by subunit *c*.

## Abstract (web summary)

Bafilomycin A1, a member of macrolide antibiotics and an autophagy inhibitor, serves as a specific and potent V-ATPases inhibitor. Here authors report the cryo-EM structure of bafilomycin A1-bound V-ATPase with six bafilomycin A1 molecules bound to the c-ring and reveal the molecular basis for Bafilomycin A1 inhibition of the V-ATPase.

## Introduction

The vacuolar-type H^+^-ATPases (V-ATPases), which are ATP-driven proton pumps responsible for modulating intracellular and extracellular pH, are involved in many physiological processes, ranging from membrane trafficking and autophagy to apoptosis, bone resorption, and sperm maturation^1–4^. Dysfunction of V-ATPase underlies diseases affecting the kidney, the central nervous system, the skin, and the skeletal muscle^5^. Genetic defects caused by mutations in V-ATPase subunits result in various syndromes, and the aberrant expression and regulation of V-ATPase is correlated with proliferation of cells from prostate, breast, lung, liver, pancreatic, and esophageal cancers^2,6^. A recent study showed that Ac45, an essential component of mammalian V-ATPase can interact with NSP6, a part of the SARS CoV-2 viral replicase/transcriptase complex implying a potential role of V-ATPase in virus infection^7^.

Mammalian V-ATPase is organized into two domains: the cytosolic *V*_1_ domain, which consists of subunits A_3_B_3_CDE_3_FG_3_H, serves as an energy source by catalyzing hydrolysis of ATP^8,9^; and the membrane-embedded *V*_o_ domain, comprised of subunits *ac*_*9*_*c″de*Ac45 and the two additional components (pro)renin receptor (PRR) and RNaseK (subunit *f*)^10–12^. The transmembrane helix 0 (TM0) of subunit *c″* along with the TMs of PRR and Ac45 constitutes the core of the *V*_o_ domain (Supplementary Fig. 1a, b). TM2 and TM4 of *c* and *c″* subunits form the outer c-ring, while their TM1 and TM3 form the inner c-ring (Supplementary Fig. 1). The *V*_1_ and *V*_o_ domains of V-ATPase can assemble and disassemble in vivo depending on various stimuli, such as growth factors, amino acid starvation, and increased glucose concentration^13,14^; therefore, the V-ATPase activity can be shut down and turned on like a switch.

**Fig. 1:**
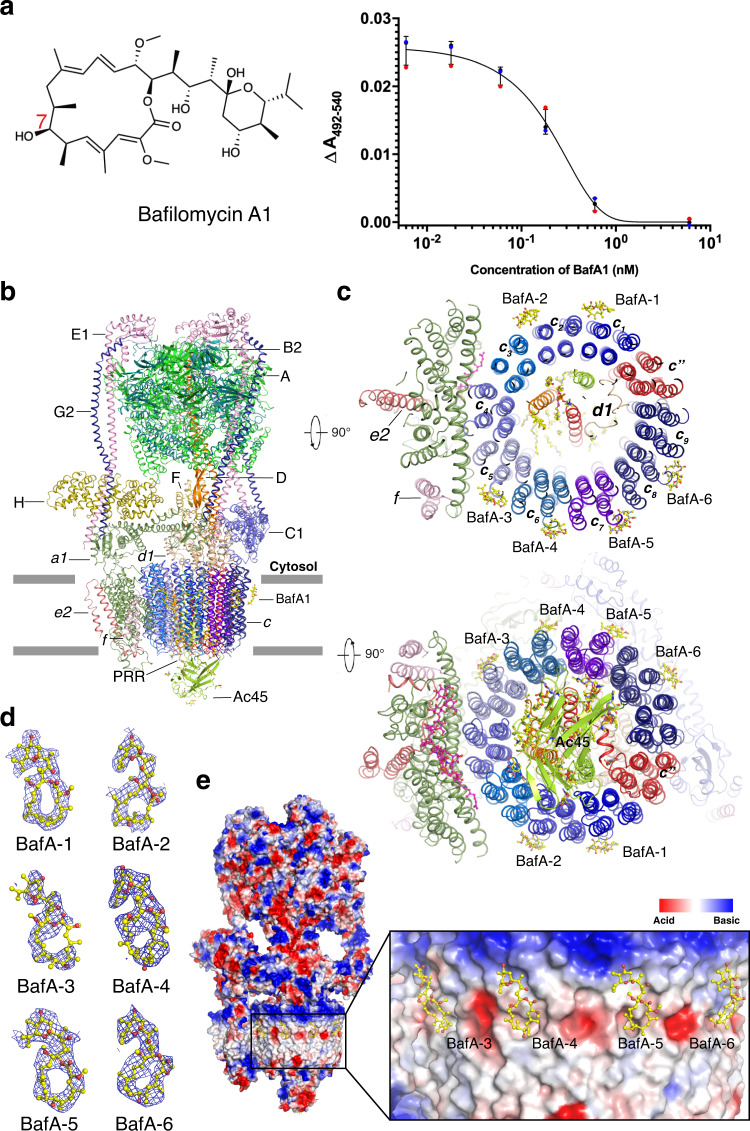
Overall structure of the V-ATPase complex with bafilomycin A1. **a** Dose-dependent ATP-driven proton translocation assay. The concentrations of bafilomycin A1 (structure shown) in the assay were 0.006 nM, 0.018 nM, 0.06 nM, 0.18 nM, 0.6 nM, and 6 nM, respectively. The concentration of V-ATPase in the assay was 0.6 nM. Data are mean ± s.d. (*n* = 3 independent experiments). The detail is described in the “Methods” section. **b** Overall structure showing bafilomycin A1 bound V-ATPase viewed from the side of the membrane. **c** The cytosolic and lumenal views of the *V*_o_ domain of V-ATPase. **d** Cryo-EM maps of each bafilomycin A1 at 4*σ* level. **e** Electrostatic surface representation of V-ATPase and bafilomycin A1 binding sites. Bafilomycin A1 is shown in yellow sticks.

Several small molecules have been identified that inhibit the activity of V-ATPase. Bafilomycin, a member of the pleomacrolides family, was identified as the first specific V-ATPase inhibitor in the 1980s (Fig. 1a). This molecule can inhibit V-ATPases from a broad range of organisms including yeast and mammals at nanomolar concentrations^15^. Intriguingly, many other V-ATPase inhibitors such as concanamycin, archazolid, and the plant toxin pea albumin 1 subunit b (PA1b) (Supplementary Fig. 2) show some overlap with the bafilomycin’s binding sites, indicating a common mechanism of inhibition^16^. Bafilomycin A1 is also widely used as a classic inhibitor of autophagy^17^. Moreover, some studies suggest that bafilomycin could serve as an anti-tumorigenic, anti-parasitic, or anti-neurodegenerative drug^18^. Because previous studies have demonstrated physiological roles of V-ATPase in the mTORC1, AMPK, Wnt, Notch, TGF-β, and GPCR signaling pathways^19–21^, inhibition of V-ATPase can also diminish cell signal transduction.

Most available inhibitors of V-ATPase exhibit cellular toxicity due to a lack of tissue specificity^22^. These inhibitors represent complex chemical structures, and their synthetic modification is challenging. Although several V-ATPase structures have been determined^10,11,23–26^, no structural evidence to date has revealed the molecular mechanism through which bafilomycin A1 or its analogs inhibits the V-ATPase. The molecular basis for how they inhibit the V-ATPase will provide valuable insights necessary for understanding the physiological role of V-ATPases and for facilitating the rational design of potential drugs.

## Results

### Overall structure of bafilomycin A1 bound V-ATPase

We purified the native V-ATPase from bovine brain according to our previously published protocol^11,27^. The resulting complex after glycerol gradient centrifugation was purified by gel filtration in the presence of 0.1% CHAPS and 0.004% glycodiosgenin (GDN). Our previous ATPase assays demonstrated that the purified endogenous V-ATPase exhibits high ATPase activity and can be inhibited by bafilomycin A1 in vitro^11^. To validate the potency of bafilomycin A1 to inhibit the V-ATPase activity, we measured the inhibitory effect of bafilomycin A1 on the proton translocation activity of V-ATPase which would be an assay for completely coupled enzyme. The result showed a nearly complete inhibition of the proton pumping activity by bafilomycin was observed at nano molar range (Fig. 1a).

We mixed the V-ATPase with bafilomycin A1 on grid preparation for cryogenic electron microscopy (cryo-EM) studies. The visualized particles were homogenous, showing clear features in the cryo-EM images, making them suitable for structural reconstruction at high resolution (Supplementary Figs. 3 and 4 and Supplementary Tables 1 and 2). Local refinement was performed by specific *V*_o_ and *V*_1_ masks improving the cryo-EM quality to show clear features of the side chains (Supplementary Figs. 3 and 5). This approach helped the unambiguous assignment of most residues and ligands. The maps after local refinement were merged by “Phenix_Combine_Focused_Map” for model building^11^. The subunits B2, C1, E1, G2, *a1*, *d1*, and *e2* have been built into the final model based on mass spectrometry results and the expression distribution in brain tissues^3^. The 3D classification enabled us to distinguish two different states as in our previous study on apo-V-ATPase^11^. However, the resolution of V-ATPase state 2 is much lower than that of state 1, the cryo-EM maps of ligand could not be distinguished. Hence, we focus our analysis and discussion of the structure on state 1 in the following text.

Six bafilomycin A1 molecules are found in the cytosolic leaflet of the c-ring (Fig. 1b, c). The average resolution of the V-ATPase *V*_o_ domain bound to bafilomycin A1 is about 3.2-Å, which is high enough for us to identify side chains and bafilomycin molecules (Supplementary Fig. 4). The bafilomycin A1 molecules show strong density when compare with the side chains near the c-ring residues at same sigma level (Supplementary Fig. 6a), and the comparison of the equilibrant areas of c-ring on cryo-EM maps of bafilomycin A1 bound V-ATPase and apo-V-ATPase side by side shows the specific binding of bafilomycin A1 molecules (Supplementary Fig. 6b). Combined with the binding model proposed by function studies^28,29^, we confirm these sites faithfully. The nine subunits *c* along with one subunit *c″* constitute the c-ring of *V*_o_ domain: remarkably, each bafilomycin A1 binds to two adjacent subunits *c* including *c*_1_*c*_2_, *c*_2_*c*_3_, *c*_5_*c*_6_*, c*_6_*c*_7_*, c*_7_*c*_8_, and *c*_8_*c*_9_ (Fig. 1c–e). Notably, bafilomycin A1 was not found in either *c*_9_*c″* or *c″c*_1_. As the binding sites in *c*_3_*c*_4_ and *c*_4_*c*_5_ have been blocked by subunit *a*, bafilomycin A1 is not able to bind these two sites. The cryo-EM maps of BafA-1, BafA-4, BafA-5, and BafA-6 are well defined, while the maps of BafA-2 and BafA-3 are not as intact as the maps of the other molecules (Fig. 1d and Supplementary Fig. 6b). We speculate that these two sites (BafA-2 and BafA-3) are close to the interface between subunit *a* and the c-ring, which may interfere with bafilomycin A1’s access to the site, causing a lower occupancy of the ligand.

### Dolichol-p-p-Glycan in *V*_o_ domain

**Fig. 2:**
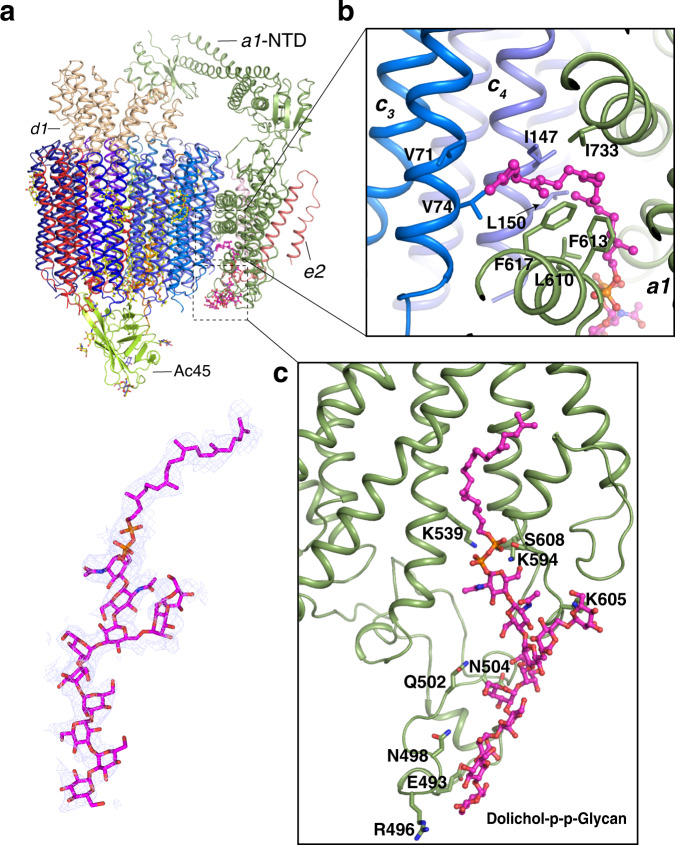
Interaction details between *V*_o_ domain and glycolipid dolichol phosphate-linked glycan (Dolichol-p-p-Glycan). **a** Overall structure showing the position of Dolichol-p-p-Glycan in Vo domain. The cryo-EM map of Dolichol-p-p-Glycan as blue mesh is shown at 3.5 σ level (lower panel). **b** The interaction details of Dolichol-p-p-Glycan chain with the *a*-CTD, *c*_3_ and c_4_ subunits. **c** The interaction details of sugar moiety of Dolichol-p-p-Glycan with the *a*-CTD. The Dolichol-p-p-Glycan is colored in magenta and the residues are shown in stick.

Recently, the remarkable studies showed the glycolipid dolichol phosphate-linked glycan (Dolichol-p-p-Glycan) can bind to the C-terminal domain (CTD) of subunit *a* partially mediating the interaction between the subunit *a* and c-ring^12,30^. Although our previous map could not identify this molecule^11^, the current map reveals the morphology of this glycolipid unambiguously supporting the previous structural and mass spectrometry identifications^12,30^; therefore, we have built it into the model (Fig. 2a). The structural analysis shows that the lipid tail of Dolichol-p-p-Glycan is engaged by *a1*-CTD and subunits *c*_3_ and *c*_4_ (Fig. 2b) and several residues of *a*1-CTD contribute the hydrophilic contacts with the sugar moiety of Dolichol-p-p-Glycan (Fig. 2c). Since the Dolichol-p-p-Glycan were found in the cryo-EM maps of yeast, rat, bovine, and human V-ATPase^10,12,30,31^, it suggests that the Dolichol-p-p-Glycan may play an important role in facilitating the proton translocation and/or the assembly of *V*_o_ domain. The specific function of Dolichol-p-p-Glycan in V-ATPase need to be further investigated.

### The interaction between bafilomycin A1 and V-ATPase

**Fig. 3:**
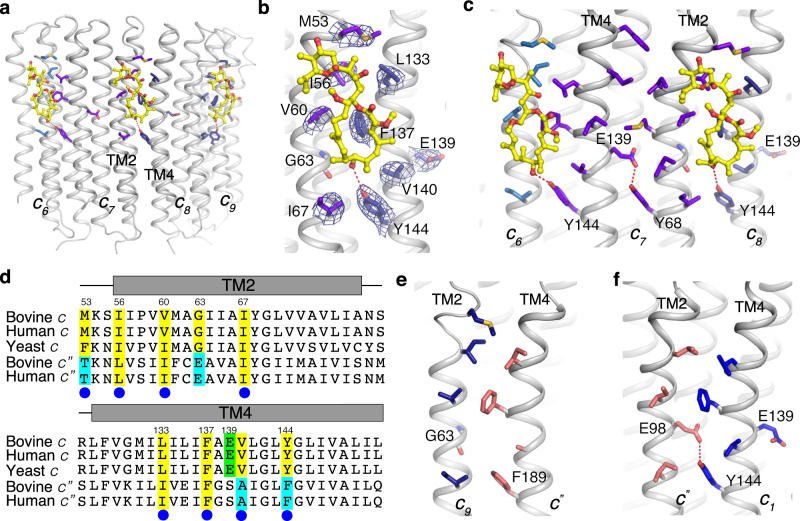
Structural details of bafilomycin A1 binding sites. **a** Overall structure showing the *c*_6_, *c*_7_, *c*_8_, and *c*_9_ subunits with bafilomycin A1. **b** The interaction details of bafilomycin A1 with the residues of the *c*_7_ and *c*_8_ subunits. The cryo-EM maps of the residues are shown at 5σ level. **c** The residue E139 confines Y68 to prevent bafilomycin A1 binding within one subunit *c* vs. binding between two adjacent subunits. **d** Sequence alignment between *c* and *c″* subunits of different species. Conserved and non-conserved residues for binding are highlighted in yellow and cyan, respectively. The catalytic residue for the proton translocation is highlighted in green. **e**, **f** Interaction details between subunit *c*″ and subunit *c*_9_ (**e**) and *c*_1_ (**f**), respectively.

The transmembrane helix 2 (TM2) of one subunit *c* and the TM4 of the neighboring subunit *c* create a composite binding site, accounting for how two subunits c engage one bafilomycin A1 molecule (Fig. 3a). Residue M53, I56, V60, and I67 of one subunit c contribute hydrophobic contacts with bafilomycin A1, while the other subunit *c* forms ligand contacts through L133, F137, V140, and Y144 (Fig. 3b). Importantly, the hydroxyl group of Y144 forms a hydrogen bond with the 7′-hydroxyl group of bafilomycin A1 (Fig. 3b). Notably, corresponding 7′-hydroxyl groups exist in archazolid A, concanamycin A, and other pleomacrolides analogs (Supplementary Fig. 2). This interaction may provide a rational why bafilomycin A1, archazolid A, and concanamycin A share a similar binding site and why they can compete with each other for binding to the *V*_o_ domain^32,33^.

Given that TM2 and TM4 are homologous and show a similar pattern of surface-exposed residues, why does bafilomycin A1 only bind to the inter-subunit site created by two adjacent *c* subunits instead of the intrasubunit space formed by TM2 and TM4 of the same subunit *c*? The structural analysis shows that the hydroxyl group of Y68 in the TM2 has been confined by a hydrophilic bond with the catalytic residue E139 (Fig. 3c). In contrast, the hydroxyl group of the analogous Y144 in TM4 is free, since the corresponding position of E139 in TM2 is G63 (Fig. 3b–d). When this glycine is mutated to Ser in yeast, the mutant strain shows a >90-fold increase in bafilomycin A1 resistance^28^. The sequence alignment between yeast, bovine, and human subunit *c* reveals that the binding residues of bafilomycin A1 are highly conserved among yeast and mammals (Fig. 3d). The mutagenesis study in fungal V-ATPase showed that mutation of the conserved binding residues (I56, L133, F137, and Y144) increased the resistance of the mutants to bafilomycin A1, supporting our structural observations^29^. Y144 of subunit *c* aligns with F189 in subunit *c*″ and hence is not conserved (Fig. 3d, e). These findings explain why the site between subunit *c*″ and subunit *c*_9_ is not utilized for bafilomycin A1 binding. Finally, Y144 of subunit *c*_1_ forms a hydrophilic interaction with E98 of subunit *c*″ TM2, rationalizing why the site between subunit *c*″ and subunit *c*_1_ also fails to engage with the bafilomycin A1 (Fig. 3f).

The structural comparison between apo and bafilomycin A1 bound bovine V-ATPase reveals that bafilomycin A1 cannot trigger the large-scale conformational changes of V-ATPase including the *V*_1_ and *V*_o_ domains (Supplementary Fig. 7). To validate this observation, we docked a bafilomycin A1 in the same position for apo state V-ATPase, neither steric hindrance nor rotamers clash was found suggesting that it is reasonable that there is no conformational change after bafilomycin binding. Notably, the structural analysis reveals that inhibition by bafilomycin A1 only functions when the c-ring rotates.

### Bafilomycin A1 employs a general mechanism to inhibit V-ATPase

**Fig. 4:**
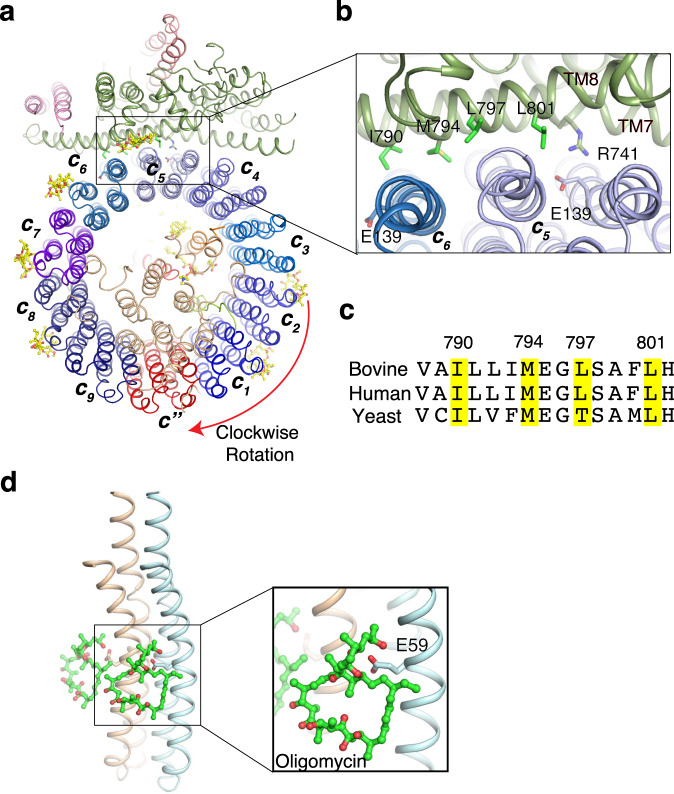
The mechanism of bafilomycin A1 inhibiting rotation of the c-ring. **a** The rotation model of the c-ring with bafilomycin A1 after a 30° rotation. **b** The residues on TM8 of subunit *a* would clash with bafilomycin A1, preventing rotation of the c-ring. **c** Sequence alignment between subunit *a* of different species. The conserved residues for clashing with bafilomycin A1 are highlighted in yellow. **d** Mechanism of oligomycin-mediated F-ATPase inhibition (PDB: 4F4S, 10.2210/pdb4F4S/pdb). Oligomycin is shown in green sticks.

ATP hydrolysis by the *V*_1_ domain can trigger rotation of the *V*_o_ domain, facilitating proton transport through the cavity between subunit *a* and c-ring^34,35^. When the c-ring rotates, bafilomycin A1 could provide steric hindrance to prevent the interactions between subunit *a* and c-ring, thereby blocking proton translocation (Fig. 4a). The residues I790, M794, L797, and L801 of subunit *a* TM8, which are highly conserved across yeast, bovine and human, clash with bafilomycin A1 (Fig. 4a, b), suggesting a general mechanism for bafilomycin A1-mediated inhibition of V-ATPase (Fig. 4c). Similarly, the crystal structure of the F-ATPase inhibitor oligomycin bound to the subunit *c* of the yeast mitochondrial ATP synthase reveals that oligomycin can block the rotation of F-ATPase c-ring^36^. These findings reveal that macrolides inhibitors share a general mechanism to inhibit the cellular rotatory machine. While oligomycin binds to the catalytic residue E59 of subunit *c* of F-ATPase (Fig. 4d), the homologous catalytic residue E139 of V-ATPase subunit *c* does not engage in bafilomycin A1 binding (Fig. 3c), suggesting differences in the detailed binding modes of macrolides inhibitors.

## Discussion

Bafilomycin A1 is the most common tool for preventing autophagy through inhibiting the activity of V-ATPase in cell biological assays^17^. The biochemistry study shows that the bafilomycin A1 inhibits ATP-driven proton translocation mediated by V-ATPase in a concentration-dependent manner (Fig. 1a). The bafilomycin A1 molecules under nanomolar show the nearly complete inhibition of proton translocation activity suggesting that these molecules could hinder the rotor rotation efficiently, which is consistent with the previous studies^37^.

Moreover, in many types of tumors, various subunits of V-ATPase are upregulated to increase cancer cell metastasis; accordingly, bafilomycin and its analogs reduce cell growth in a number of cancer cell lines by inducing cell apoptosis^1^. However, the clinical use of these V-ATPase inhibitors is limited by their substantial tissue toxicity^22^, since V-ATPase is a housekeeping enzyme. Here, we report the structure of bafilomycin A1 bound V-ATPase from a mammalian brain, which informs about potential modifications of inhibitors for the treatment of human disease. Decreasing the binding affinity of bafilomycin A1 to V-ATPase from nanomolar to sub-micromolar affinity, for example, may reduce binding to low-abundance V-ATPase under physiological conditions but allow the compound to bind to upregulated V-ATPase in a tumor environment. Finally, this structure may facilitate the design of a fungal V-ATPase-specific inhibitor used as an antibiotic for the treatment of fungal infections.

## Methods

### Gain clathrin-coated vesicles

The bovine brains were purchased from Animal Technologies. Three defatted bovine brains were rinsed and blended with buffer A containing 0.1 M Na-MES, pH 6.5, 1 mM EGTA, 0.5 mM MgCl_2_, and 3 mM NaN_3_. One kilogram of the tissue was homogenized in 900 ml of buffer A. The homogenate was centrifuged in a GSA (Sorvall) rotor at 20,000×*g* for 50 min and the resulting supernatant was further centrifuged at 140,000×*g* for 1 h to sediment the membrane vesicles. The pellet containing enriched coated vesicles was collected and resuspended in buffer A at a protein concentration of 15 mg/ml, frozen in liquid nitrogen, and stored at −80 °C for the future experiments.

### Purification of V-ATPase

The V-ATPase was purified according to the previously described protocol^27^. Briefly, the membrane vesicles were incubated with 0.75 M Tris-HCl pH 8.0 on ice for 30 min to strip clathrin. After centrifugation at 150,000×*g* for 35 min, the pellet was resuspended in 0.5% sodium cholate and incubated at 0 °C for 30 min. After centrifuge at 150,000×*g* for 35 min, the pellet was resuspended in 0.75% (v/v) C_12_E_9_, 10 mM Tris-MES pH 6.75, incubated on ice for 60 min and centrifuged at 150,000×*g* for 90 min. The supernatant was applied to a hydroxyapatite column which had been equilibrated with buffer B containing 0.1% (v/v) C_12_E_9_, 10% (v/v) glycerol, 0.5 mM DTT, 10 mM Tris-MES pH 7.0. The column was washed with 2 CV of buffer B and eluted with buffer B containing 0–0.3 M Na-phosphate. Saturated ammonium sulfate solution was added dropwise to the active hydroxyapatite fractions (selected by either SDS-PAGE or ATPase assay), to a final concentration of 1.65 M. The mixture was centrifuged at 100,000×*g* for 30 min, the pellet was harvest and dissolved in buffer containing 0.1% (v/v) C_12_E_9_, 0.5 mM DTT and 10 mM Tris-MES pH 7.0. The resulting sample was loaded on to a glycerol gradient (12 ml, 10–30%) prepared in buffer B. The gradient was centrifuged at 170,000×*g* for 20 h and fractions were collected. The fractions containing V-ATPase were concentrated and further purified by gel filtration using a Superose 6 10/300 column (GE Healthcare) pre-equilibrated with buffer C containing 20 mM Hepes pH 7.5, 150 mM NaCl, 0.1% CHAPS, 0.004% glyco-diosgenin (Anatrace). The peak fractions were collected and concentrated to ~3 mg/ml for cryo-EM grid preparation.

### Reconstitution of V-ATPase into proteoliposomes

The bovine brain V-ATPase was reconstituted into proteoliposomes, which contain phosphatidylcholine (PC), phosphatidylethanolamine (PE), Phosphatidylserine (PS), and cholesterol at a weight ratio of 40:26.5:7.5:26, by the cholate dilution, freeze-thaw method, as described^38^. In brief, the pure lipids, dissolved in chloroform, were mixed at the ratios, and were evaporated under N_2_, then the lipids were further dried under reduced pressure for 1 h and dissolved in buffer with 10 mM Tris-MES pH 7.0, 1% sodium cholate, 1 mM DTT to yield a final liposome concentration of 50 mg/ml. Liposomes (300 μg) were added to 1 μg of V-ATPase and were well mixed for each reaction. Glycerol, sodium cholate, KCl, MgCl_2_, and DTT were added to the protein-lipid mixture at final concentrations of 10% (v/v), 1%, 150 mM, 2.5 mM, and 2 mM respectively. The reconstitution mixtures were incubated at room temperature for 1 h, frozen in liquid N_2_ for 15 min, and then thawed on the ice, which can be used after 4 h.

### Measurement of ATP-driven proton translocation assay

Generally, for each reaction, 30 μl of proteoliposomes with DMSO/bafilomycin A1 were added to 1.6 ml of proton-pumping assay buffer containing 10 mM Tricine, pH 7.0, 6.7 μM acridine orange, 3 mM MgCl_2_, and 150 mM KCl. The reaction was initiated by addition of 1.3 mM ATP pH 7.0, 1 μg/ml valinomycin. The assay was conducted in a SLM-Aminco DW2C dual wavelength spectrophotometer and the activity was registered as ΔA_492-540_ as described^39^. The concentrations of bafilomycin A1 in the assay were 0.006 nM, 0.018 nM, 0.06 nM, 0.18 nM, 0.6 nM and 6 nM, respectively. The concentrations of V-ATPase in the assay was 0.6 nM. The experiment has been repeated twice, and the result was performed using GraphPad Prism8.

### EM sample preparation and imaging for Cryo-TEM

For preparation of bafilomycin A1-V-ATPase complex sample, freshly purified V-ATPase (final concentration 3 μM) in buffer C was mixed with bafilomycin A1(final concentration 30 μM) for 30 min prior to grid preparation, then the sample was applied to Quantifoil R1.2/1.3 400 mesh Au holey carbon grids (Quantifoil). The grids were then blotted and plunged into liquid ethane for flash freezing using a Vitrobot Mark IV (FEI). The grids were imaged in a 300 keV Titan Krios (FEI) with a Gatan K3 Summit direct electron detector (Gatan) in super resolution mode using the data collection software Serial EM. Dark-subtracted images collected at super-resolution mode were first normalized by gain reference and binned two-fold, which resulted in a pixel size of 0.833 Å with a counted rate of 23 electrons per physical pixel per second^40^. Images were recorded for 1.8 s exposures in 60 subframes with a total dose of 60 electrons per Å2 and a defocus range of −1.0 to −2.0 μm.

### Imaging processing and 3D reconstruction for Cryo-TEM

The images used for this session were divided into two parts for data processing. Dark subtracted images were first normalized by gain reference and then binned twofold that resulted in a pixel size of 0.833 Å. Motion correction and gain reference was performed using the program MotionCor2^41^. The contrast transfer function (CTF) was estimated using CTFFIND4^42^. The low-quality images and false-positive particles were removed manually. The V-ATPase templates for automatic picking was from the apo-V-ATPase data^11^, and after auto-picking, about 149K/77K particles of V-ATPase were extracted, then the particles were classified by 2D classification in RELION-3^43^. We used the cryo-EM structure of apo-V-ATPase which was determined by us with the data collected from a 300 keV Krios (FEI) low-pass filtered to 40 Å as the initial model and apply a mask for 3D classification. The best class of these two parts, containing 30,232/10,377 particles, were combined together, and provided a 3.93 Å map after 3D auto-refinement with a mask and postprocess in RELION-3. Then the second 3D classification was performed with six classes. Two rotational states were separated by the obvious position of subunits D and F. The class of state 1 including 26,530 particles provided a 3.93 Å map after 3D auto-refinement with a mask and postprocess in RELION-3. The class of state 2 including 12,733 particles provided a 4.26 Å map after 3D auto-refinement with a mask and postprocess in RELION-3. For both state 1 and state 2, the CTF refinement and Bayesian polishing of particles were performed using RELION-3 followed by 3D refinement using a soft mask. Applying a soft mask in RELION-3 post-processing yielded a final cryo-EM map of 3.62 Å for state 1 and 4.17 Å for state 2. Resolution was estimated using the Fourier shell correlation (FSC) 0.143 criterion by RELION-3.

For rotational state1, after Bayesian polishing and 3D refinement, applying a full mask in RELION-3 post-processing yielded a 3.79 Å overall resolution based on the Fourier shell correlation (FSC) 0.143 criterion. But due to the flexibility of *V*_1_ and *V*_o_, *V*_o_ were not well resolved. We referred to our previously method, and several focused refinements with different masks were attempted to get a high-quality local map. These focused refinements included subunits A_3_B_3_DE_3_FG_3_, subunits CFHV_o_, c ring region provided some good local maps, especially for the *V*_o_ part (Supplementary Figs. 3 and 4). After postprocessing, these refinements gave a resolution of 3.39 Å, 3.83 Å, and 3.47 Å, respectively, then the CTF refinement were performed using RELION-3 followed by 3D refinement using the local masks. After postprocessing, these refinements gave a resolution of 3.20 Å, 3.59 Å, and 3.25 Å, respectively. All these focused maps can be used to generate a composite map for refinement.

### Model construction, model refinement and validation

To obtain better side-chain densities for model building, we sharpened the map of state1 of bafilomycin A1 bound V-ATPase using post-processing in RELION-3 with a B-factor value of −64.4 Å^2^. The atomic model of bafilomycin A1 bound V-ATPase was based on the structure of apo-V-ATPase (PDB: 6XBW)^11^. The overall structure of state 1 was docked into the cryo-EM map of bafilomycin A1 bound V-ATPase using Chimera^44^ to generate the initial model, and then manually adjusted using COOT^45^. The residues of bovine V-ATPase that were not resolved or built were shown in Supplementary Table 1.

For bafilomycin A1 bound V-ATPase complex, to generate a composite map for refinement, several focused maps that have been described above were combined and aligned using phenix.combine_focused _maps, which would coalescence the best parts of several maps together. The model was refined in real space using PHENIX^46^ with secondary-structure restraints and stereochemical restraints^47^. For cross-validations, the final model was refined against one of the half maps generated by 3D auto-refine and the model vs. map FSC curves were generated in the comprehensive validation module in PHENIX. MolProbity^48^, EMRinger^49^, *Q*-score^50^ and PHENIX were used to validate the final model. Local resolutions were estimated using RELION-3. Structure figures were generated using PyMOL (http://www.pymol.org), Chimera^44^, and ChimeraX^51^.

### Reporting summary

Further information on research design is available in the Nature Research Reporting Summary linked to this article.

## Supplementary information

Supplementary Information

Reporting Summary

## Data Availability

Data supporting the findings of this manuscript are available from the corresponding authors upon reasonable request. A reporting summary for this Article is available as a Supplementary Information file. The 3D cryo-EM density map of bafilomycin A1 bound V-ATPase has been deposited in the Electron Microscopy Data Bank under the accession numbers EMD-22880. Atomic coordinate for the atomic model of bafilomycin A1 bound V-ATPase has been deposited in the Protein Data Bank under the accession numbers PDB 7KHR [10.2210/pdb7KHR/pdb] . Source data are provided with this paper.

## Source data

Source Data
