# Supplementary material for: Molecular basis of V-ATPase inhibition by bafilomycin A1
Source: Nat Commun. 2021 Mar 19;12:1782. doi: 10.1038/s41467-021-22111-5 (PMC7979754; doi:10.1038/s41467-021-22111-5)
Supplement: Supplementary file 1 — Supplementary Information [file 41467_2021_22111_MOESM1_ESM.pdf]

Supplementary Information for

Molecular Basis of V-ATPase Inhibition by Bafilomycin A1

Rong Wang, Jin Wang, Abdirahman Hassan, Chia-Hsueh Lee, Xiao-Song Xie*, and Xiaochun Li*

*Correspondence to Xiao-Song.Xie@UTSouthwestern.edu or Xiaochun.Li@UTSouthwestern.edu

This PDF file includes:

Supplementary Figs. 1-7
Supplementary Tables 1 and 2

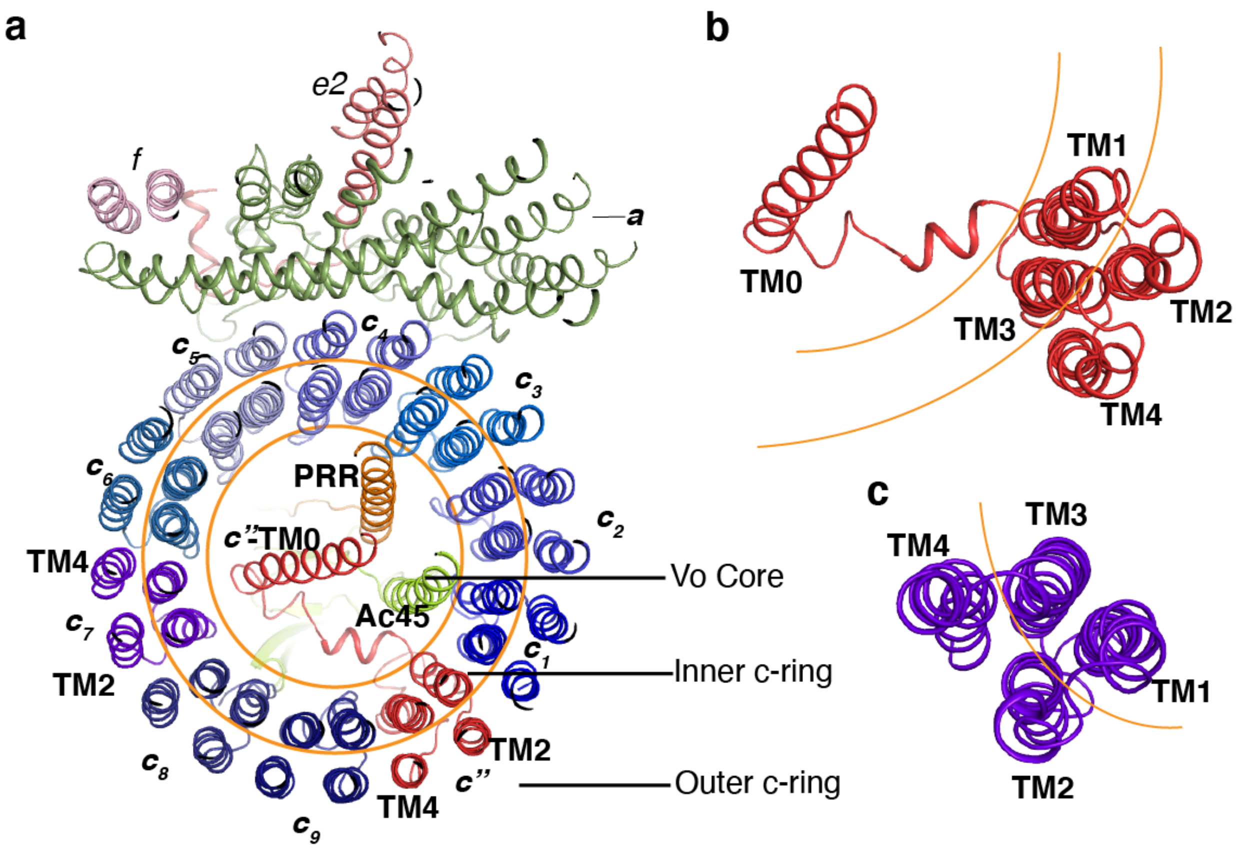

Supplementary Fig. 1 Structure of bovine V-ATPase V_o domain.

a. Cytosolic view of the V_o domain. Each subunit is labeled. The core domain of the c-ring contains TM0 of subunit *c"* and TMs of PRR and Ac45. TM1 and TM3 of subunit *c* and *c"* form the inner c-ring, while TM2 and TM4 of subunit *c* and *c"* form the outer c-ring. **b.** Cytosolic view of subunit *c"*. **c.** The cytosolic view of subunit *c*. Each TM is labeled.

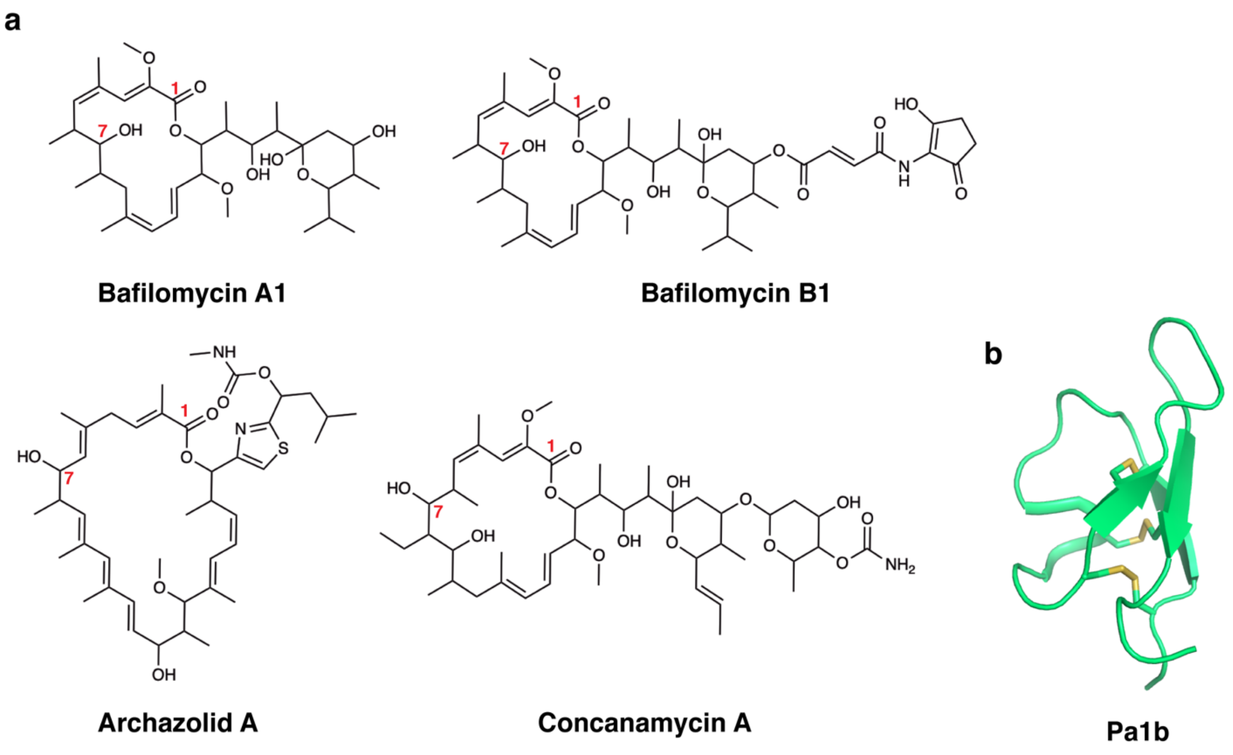

Supplementary Fig. 2 The inhibitors of V-ATPase.

a. The chemical structures of macrolides family. The 7'-hydroxyl group of each compound is indicated. **b.** The NMR structure of pea albumin 1, subunit b (PA1b) (PDB code: 1P8B, http://doi.org/10.2210/pdb1P8B/pdb). The disulfide bonds of PA1b are shown in sticks.

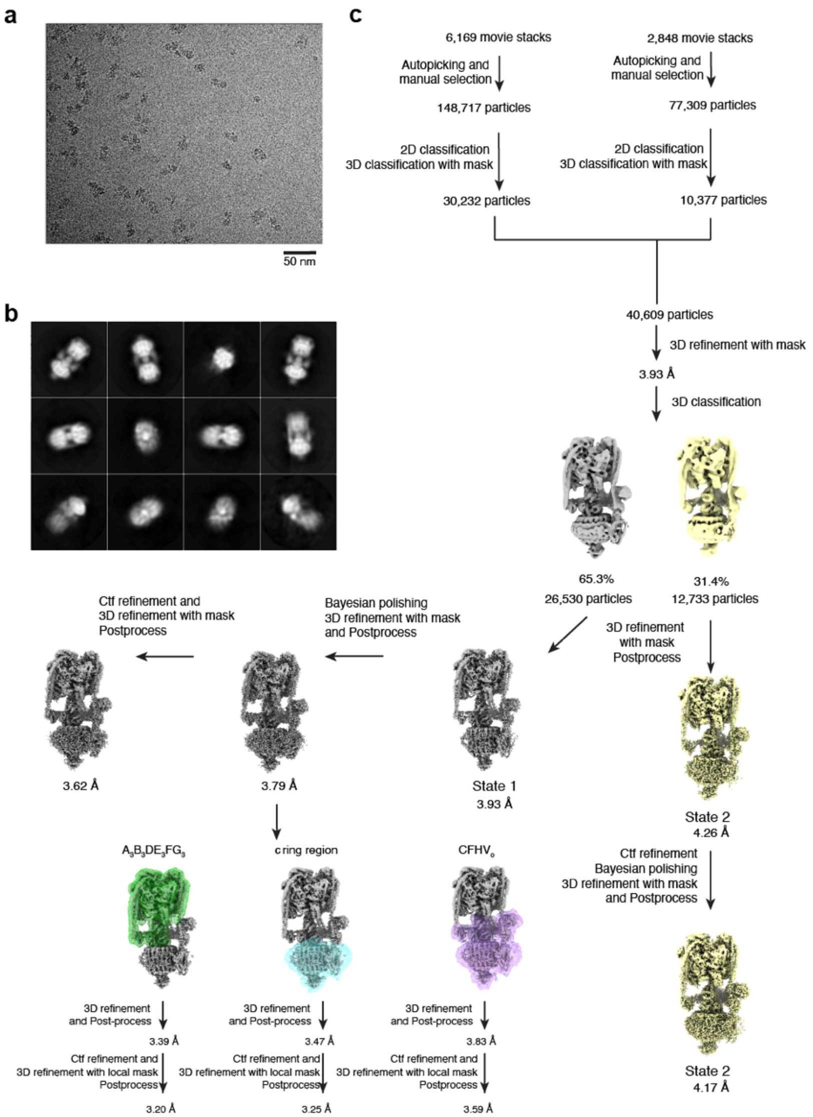

Supplementary Fig. 3 Data processing.

a. A representative electron micrograph at -2.0 μm defocus. **b.** The cryo-EM 2D classification from RELION is shown. **c.** The data processing work-flow.

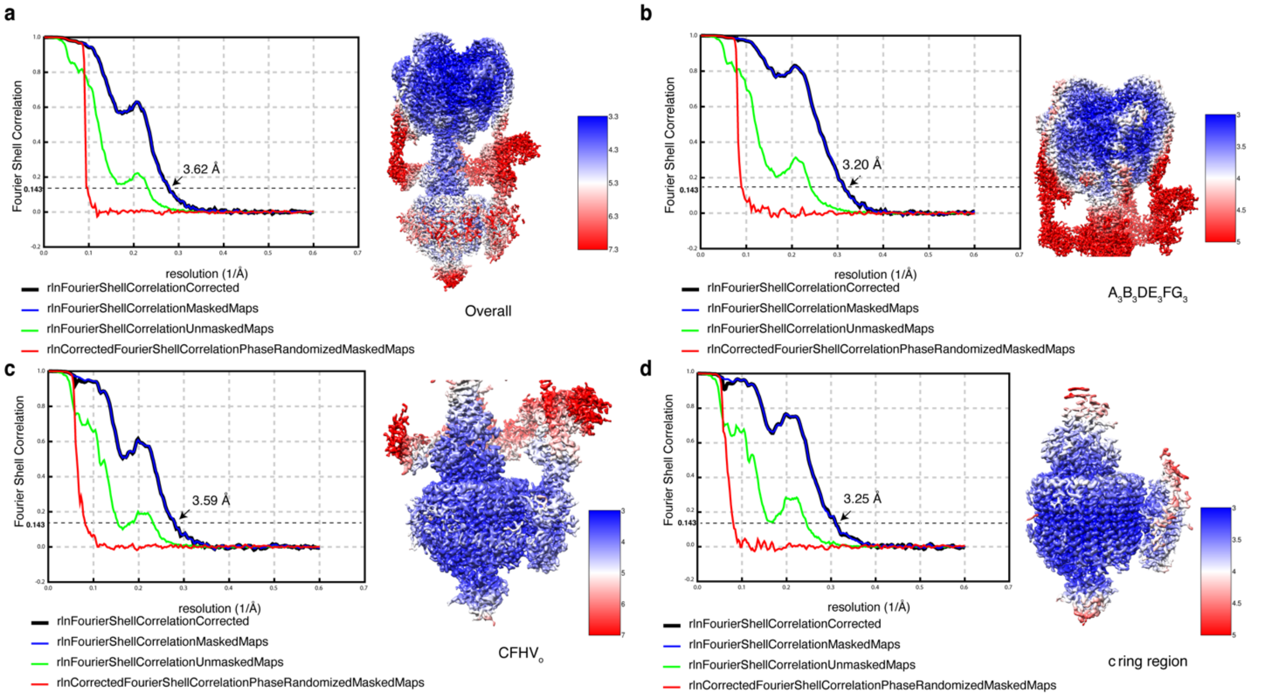

Supplementary Fig. 4 FSC curve and local resolution estimation.

a. Fourier shell correlation (FSC) curve as a function of resolution of intact bafilomycin A1-V-ATPase using RELION-3 output and density maps colored by local resolution estimation using RELION-3. **b.** Fourier shell correlation (FSC) curve as a function of resolution of $A_3B_3DE_3FG_3$ using RELION-3 output and density maps colored by local resolution estimation using RELION-3. **c.** Fourier shell correlation (FSC) curve as a function of resolution of $CFHV_o$ using RELION-3 output and density maps colored by local resolution estimation using RELION-3. **d.** Fourier shell correlation (FSC) curve as a function of resolution of c-ring using RELION-3 output and density maps colored by local resolution estimation using RELION-3.

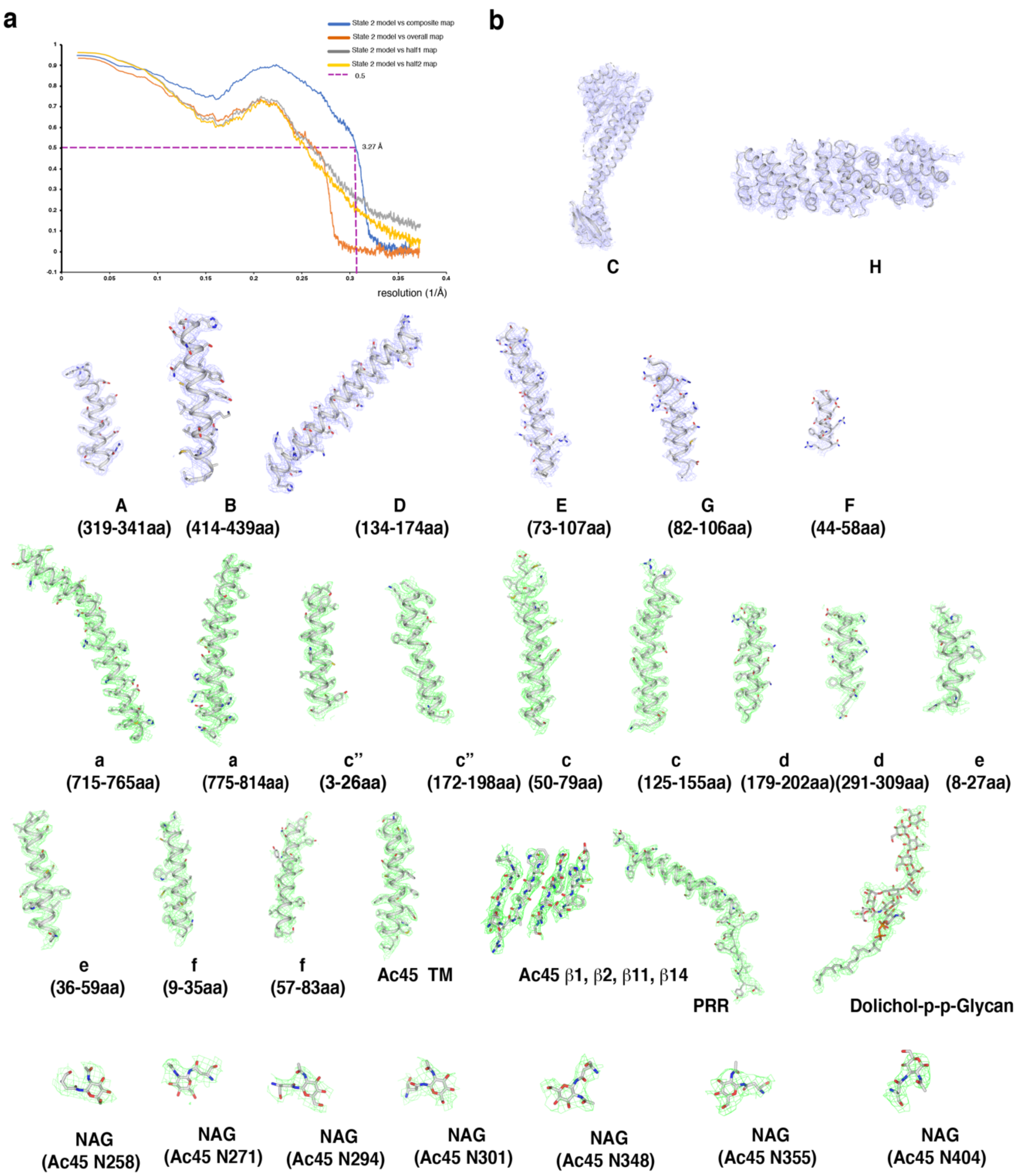

Supplementary Fig. 5 Cryo-EM map of structural elements.

a. The FSC curves of calculated between the refined structure model and the composite map (blue) used for refinement, the overall half map (gray), the other half map (yellow) and the overall full map (orange). **b.** The structural elements of bafilomycin A1-bound V-ATPase complex with cryo-EM map.

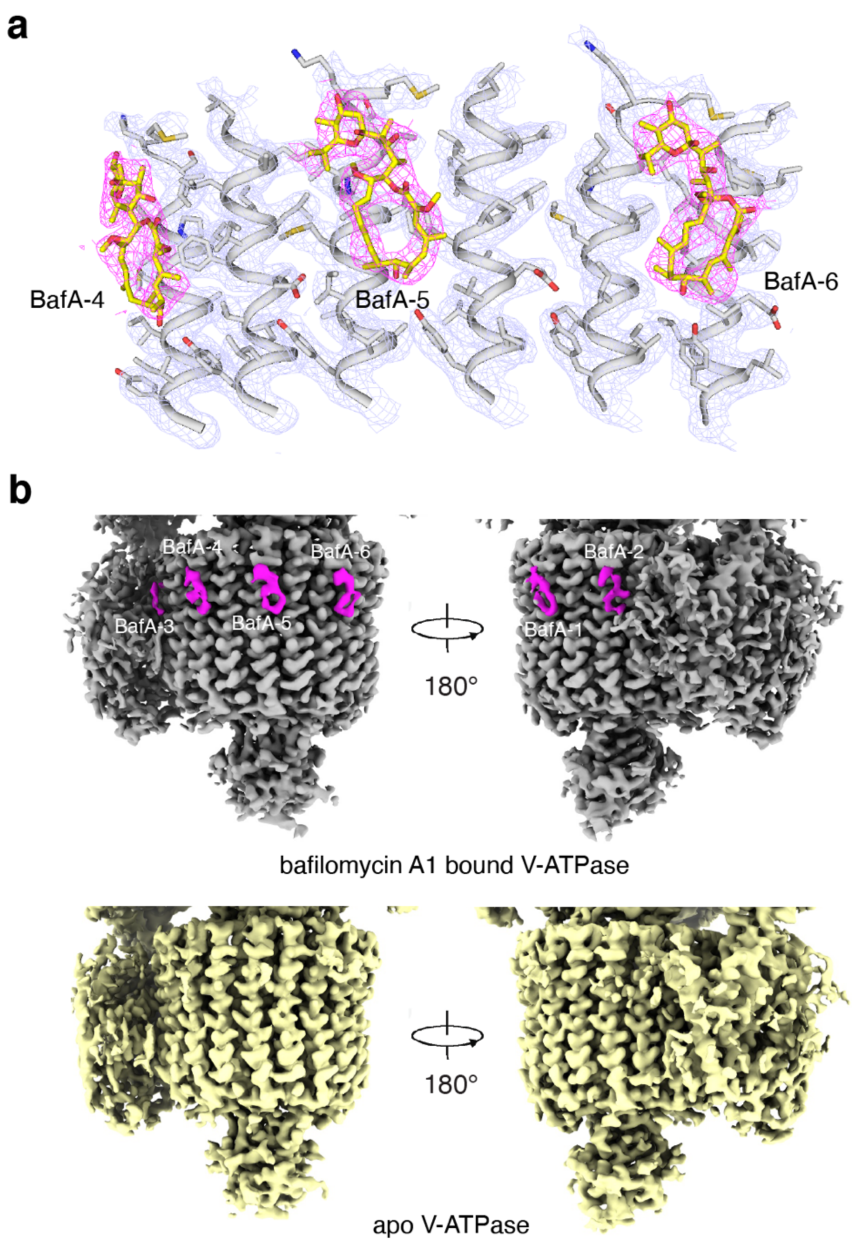

Supplementary Fig. 6 Comparison of bafilomycin A1 bound V-ATPase and apo-V-ATPase revealing the specific binding sites of bafilomycin A1 (BafA).

a. Cryo-EM map for BafA-4, BafA-5 and BafA-6. C-ring and bafilomycin A1 are shown at the same level of 4σ. **b.** Cryo-EM maps of bafilomycin A1 bound V-ATPase and apo-V-ATPase. The equilibrant areas of bafilomycin A1 binding in the apo state are shown compared with that in the bafilomycin A1 bound V-ATPase.

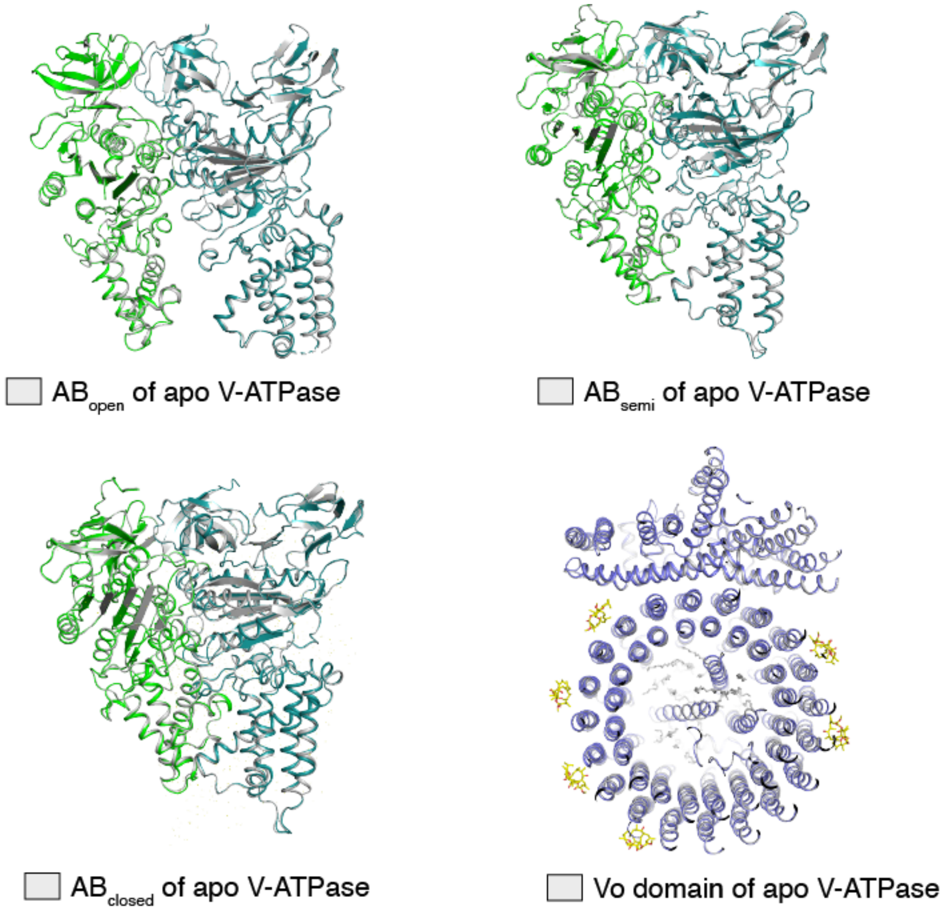

Supplementary Fig. 7 Structural comparison of apo and bafilomycin A1-bound V-ATPase.

Structural comparison of the AB subunits and V_o domain in apo and bafilomycin A1-bound bovine V-ATPase.

Supplementary Table 1. Summary of each subunit of bafilomycin A1-V-ATPase complex model.

Subunit	Chain	Total Residues Built	Remove Side Chains	Unmodelled
A	A	16-612		1-15, 558-565, 613-617
A	B	15-612		1-14, 251-257, 613-617
A	C	13-612		1-12, 613-617
B	D	38-506		1-37, 217-224, 507-511
B	E	38-506		1-37, 214-224, 507-511
B	F	38-506		1-37, 214-224, 507-511
C	G	2-379	all	1, 12-17, 81-85, 345-365, 380-382
D	H	6-217		1-5, 218-247
E	I	6-223	6-70	1-5, 224-226
E	J	6-223	6-47	1-5, 224-226
E	K	4-223	4-66	1-3, 224-226
F	L	5-107		1-4, 108-119
G	M	6-114	6-76	1-5, 115-118
G	N	6-114	6-66	1-5, 115-118
G	O	6-114	6-70	1-5, 115-118
H	P	66-445	66-220, 333-355	1-65, 108-119, 446-476
a	a	4-834	167-245	1-3, 141-166, 659-713, 835-838
c’’	b	2-205		1
d	d	4-351		1-3
e	e	8-80		1-7, 81
f	f	9-83		1-8, 46-55, 84-98
c	c	6-155		1-5
c	g	6-155		1-5
c	k	6-155		1-5
c	l	6-155		1-5
c	m	6-155		1-5
c	n	6-155		1-5
c	o	5-155		1-4
c	p	5-155		1-4
c	q	5-155		1-4
PRR	r	293-338		1-292, 339-351
Ac45	s	252-455		1-251, 456-468

Supplementary Table 2. Cryo-EM data collection, refinement and validation statistics.

	Bafilomycin A1-V-ATPase complex (EMDB-EMD-22880) (PDB-7KHR)
**Data collection and processing**	
Magnification	60024
Voltage (kV)	300
Electron exposure (e–/Å^2)	60
Defocus range (μm)	1.0 to 2.0
Pixel size (Å)	0.833
Symmetry imposed	C1
Initial particle images (no.)	226,026
Final particle images (no.)	26,530
Overall map resolution (Å)	3.6
FSC threshold	0.143
**Refinement**	
Initial model used (PDB code)	6XBW
Model resolution (Å)	3.27
FSC threshold	0.5
Map sharpening B factor (Å^2)	-64.4
Model composition	
Non-hydrogen atoms	61913
Protein residues	8212
Ligands	36
B factors (Å^2)	
Protein	53.89
Ligand	49.63
R.m.s. deviations	
Bond lengths (Å)	0.006
Bond angles (°)	1.014
Validation	
Q-score	Protein: 0.591<br>BAF: 0.666<br>(Expected Q@3.62.=0.48)
MolProbity score	1.64
EMRinger score	2.61 (4255 side chains)
Clashscore	5.82
Poor rotamers (%)	0.27
Ramachandran plot	
Favored (%)	95.32
Allowed (%)	4.64
Disallowed (%)	0.04
