# Supplementary material for: Molecular basis of V-ATPase inhibition by bafilomycin A1
Source: Nat Commun. 2021 Mar 19;12:1782. doi: 10.1038/s41467-021-22111-5 (PMC7979754; doi:10.1038/s41467-021-22111-5)
Supplement: Supplementary file 2 — Reporting Summary [file 41467_2021_22111_MOESM2_ESM.pdf]

nature research

Corresponding author(s): Xiaochun Li

Last updated by author(s): Jan 28, 2021

Reporting Summary

Nature Research wishes to improve the reproducibility of the work that we publish. This form provides structure for consistency and transparency in reporting. For further information on Nature Research policies, see Authors & Referees and the Editorial Policy Checklist .

Statistics

For all statistical analyses, confirm that the following items are present in the figure legend, table legend, main text, or Methods section.

n/a	Confirmed	
☐	☒	The exact sample size (*n*) for each experimental group/condition, given as a discrete number and unit of measurement
☐	☒	A statement on whether measurements were taken from distinct samples or whether the same sample was measured repeatedly
☒	☐	The statistical test(s) used AND whether they are one- or two-sided<br>*Only common tests should be described solely by name; describe more complex techniques in the Methods section.*
☒	☐	A description of all covariates tested
☒	☐	A description of any assumptions or corrections, such as tests of normality and adjustment for multiple comparisons
☐	☒	A full description of the statistical parameters including central tendency (e.g. means) or other basic estimates (e.g. regression coefficient) AND variation (e.g. standard deviation) or associated estimates of uncertainty (e.g. confidence intervals)
☒	☐	For null hypothesis testing, the test statistic (e.g. *F*, *t*, *r*) with confidence intervals, effect sizes, degrees of freedom and *P* value noted<br>*Give P values as exact values whenever suitable.*
☒	☐	For Bayesian analysis, information on the choice of priors and Markov chain Monte Carlo settings
☒	☐	For hierarchical and complex designs, identification of the appropriate level for tests and full reporting of outcomes
☒	☐	Estimates of effect sizes (e.g. Cohen's *d*, Pearson's *r*), indicating how they were calculated

Our web collection on statistics for biologists contains articles on many of the points above.

Software and code

Policy information about availability of computer code

Data collection	SerialEM3.7.10
Data analysis	CTFFIND4 4.1.8, Relion 3.0, BFactor 1.04, Refmac 5.8.0091, COOT 0.8.8, Phenix 1.14, PyMOL 1.8.6.0, MolProbity 4.3, Chimera 1.12, MotionCor2 1.2.1, GraphPad Prism8, Chimera X, Espript 3.0, EMRinger v1.0.0

For manuscripts utilizing custom algorithms or software that are central to the research but not yet described in published literature, software must be made available to editors/reviewers. We strongly encourage code deposition in a community repository (e.g. GitHub). See the Nature Research guidelines for submitting code & software for further information.

Data

Policy information about availability of data

All manuscripts must include a data availability statement. This statement should provide the following information, where applicable:

- Accession codes, unique identifiers, or web links for publicly available datasets
- A list of figures that have associated raw data
- A description of any restrictions on data availability

The 3D cryo-EM density map of bafilomycin A1-bound V-ATPase has been deposited in the Electron Microscopy Data Bank under the accession numbers EMD-22880. Atomic coordinate for the atomic model of bafilomycin A1-bound V-ATPase has been deposited in the Protein Data Bank under the accession numbers 7KHR. All other data is available from the corresponding authors upon reasonable request.

Field-specific reporting

Please select the one below that is the best fit for your research. If you are not sure, read the appropriate sections before making your selection.

- [x] Life sciences
- [ ] Behavioural & social sciences
- [ ] Ecological, evolutionary & environmental sciences

For a reference copy of the document with all sections, see nature.com/documents/nr-reporting-summary-flat.pdf

Life sciences study design

All studies must disclose on these points even when the disclosure is negative.

Sample size	Sample size was determined for obtaining enough number of particles to reconstitute obtain high quality 3D map.
Data exclusions	No data was excluded.
Replication	Each experiment was reproduced at least two times on separate occasions. All attempts at replication were successful.
Randomization	Randomization was not necessary as the independent variables to be tested were sufficient for the functional interpretations within this study.
Blinding	No experimental group allocation was carried out. Blinding is not necessary or valid for the purposes of structural determination.

Reporting for specific materials, systems and methods

We require information from authors about some types of materials, experimental systems and methods used in many studies. Here, indicate whether each material, system or method listed is relevant to your study. If you are not sure if a list item applies to your research, read the appropriate section before selecting a response.

Materials & experimental systems

n/a	Involved in the study	
x		Antibodies
x		Eukaryotic cell lines
x		Palaeontology
	x	Animals and other organisms
x		Human research participants
x		Clinical data

Methods

n/a	Involved in the study	
x		ChIP-seq
x		Flow cytometry
x		MRI-based neuroimaging

Animals and other organisms

Policy information about studies involving animals; ARRIVE guidelines recommended for reporting animal research

Laboratory animals	N.A.
Wild animals	The wild-type cow brains were purchased from Animal Technologies.
Field-collected samples	N.A.
Ethics oversight	No ethical approval or guidance was required since the tissues were purchased from a company directly.

Note that full information on the approval of the study protocol must also be provided in the manuscript.
